# Supplementary figures and images for: Recurrent Interneuron Connectivity Does Not Support Synchrony in a Biophysical Dentate Gyrus Model
Source: eNeuro. 2025 Apr 18;12(4):ENEURO.0097-25.2025. doi: 10.1523/ENEURO.0097-25.2025 (PMC12017885; doi:10.1523/ENEURO.0097-25.2025)

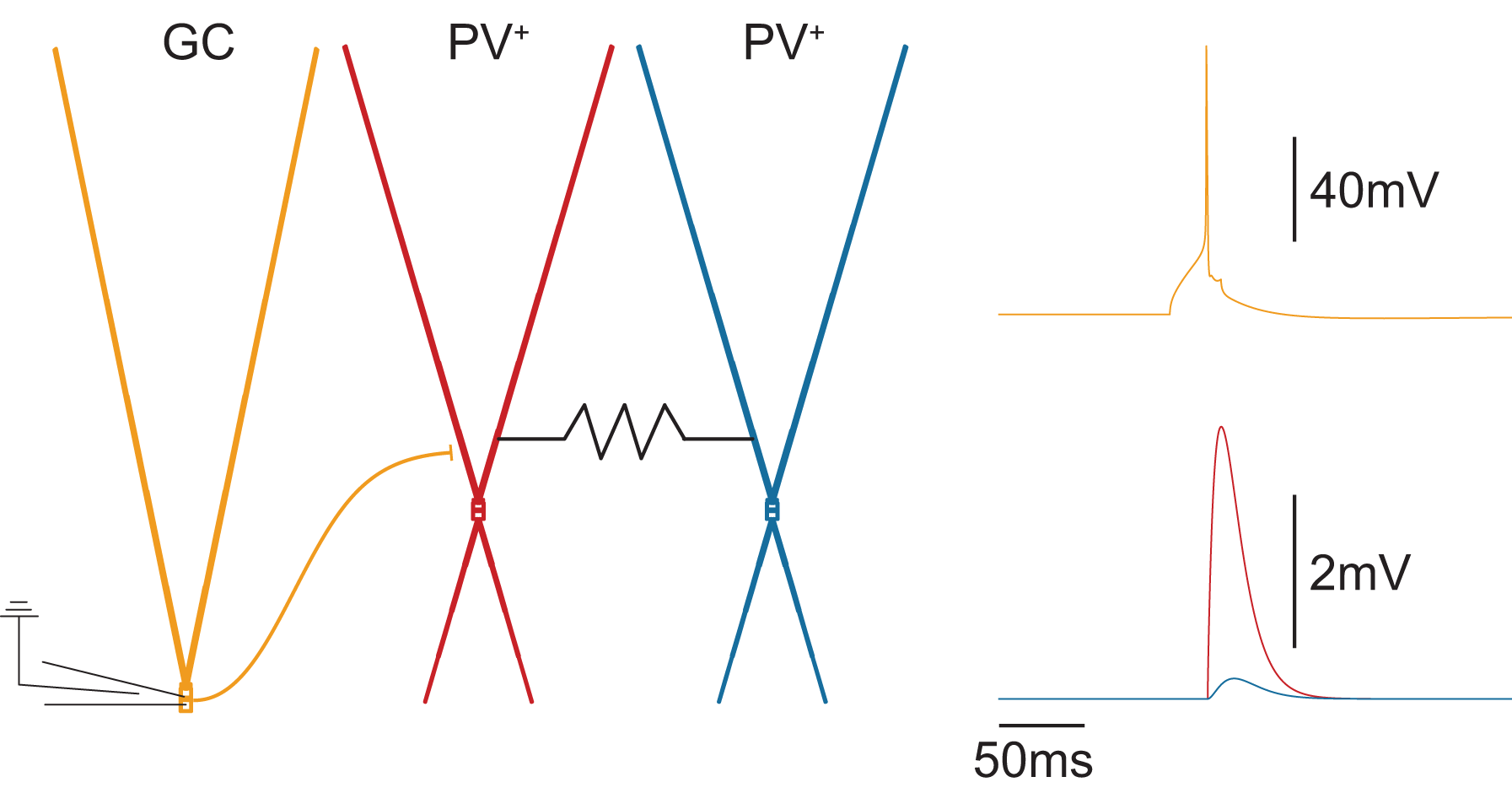

Supplement: Figure 1-1 — Granule cell EPSP is measurable in a coupled PV+ IN. Gap junction resistance and placement as in Figure 1 B. Download Figure 1-1, TIF file. [file eneuro-12-ENEURO.0097-25.2025-s002.tif]

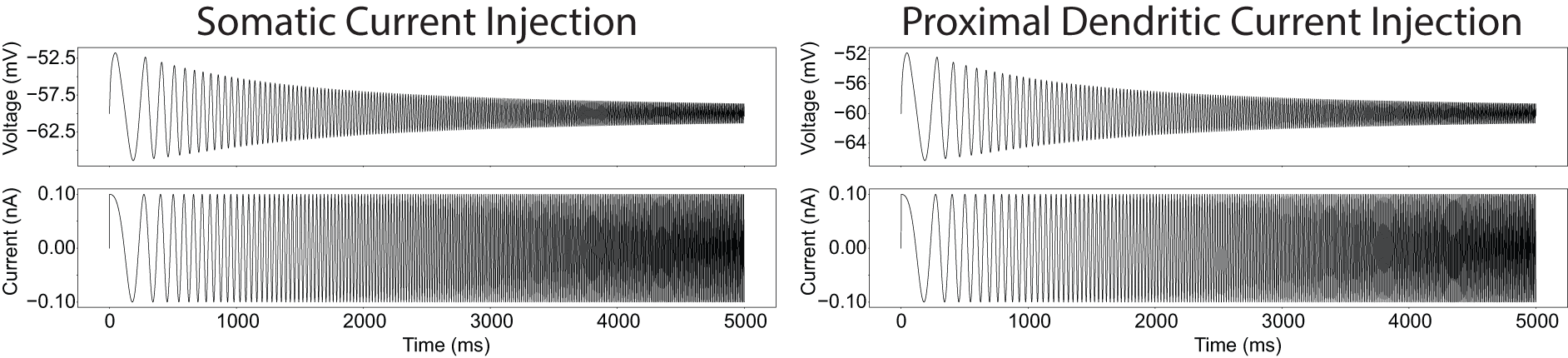

Supplement: Figure 1-2 — The PV+ IN model does not resonate below threshold. The bottom shows a chirp current that is injected into the neuron. The frequency of the chirp current rises linearly from 1 Hz to 100 Hz over 5 s. The top shows the voltage response to the current as measured at the soma. On the left, the current was injected into the soma. On the right, the current was injected into a randomly chosen proximal dendritic segment. Download Figure 1-2, TIF file. [file eneuro-12-ENEURO.0097-25.2025-s003.tif]

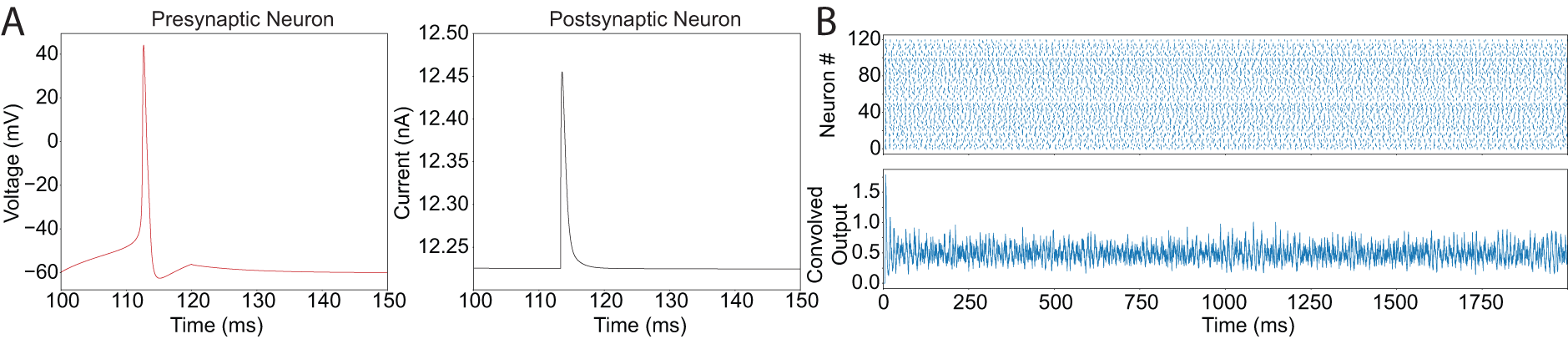

Supplement: Figure 1-3 — Doubling the strength of the PV-PV synapse does not induce synchrony with biologically plausible connectivity. A shows the strenght of the PV-PV synapse as it is used throughout the paper. The postsynaptic neuron is voltage clamped to 0 mV. The peak amplitude of the IPSC is 229 pA. That is larger than most IPSCs measured by Espinoza et al. (2018). B shows the spiking activity in the PV+ ring network with doubled synaptic conductance (15.2 nS) and biologically plausible connectivity. Download Figure 1-3, TIF file. [file eneuro-12-ENEURO.0097-25.2025-s004.tif]

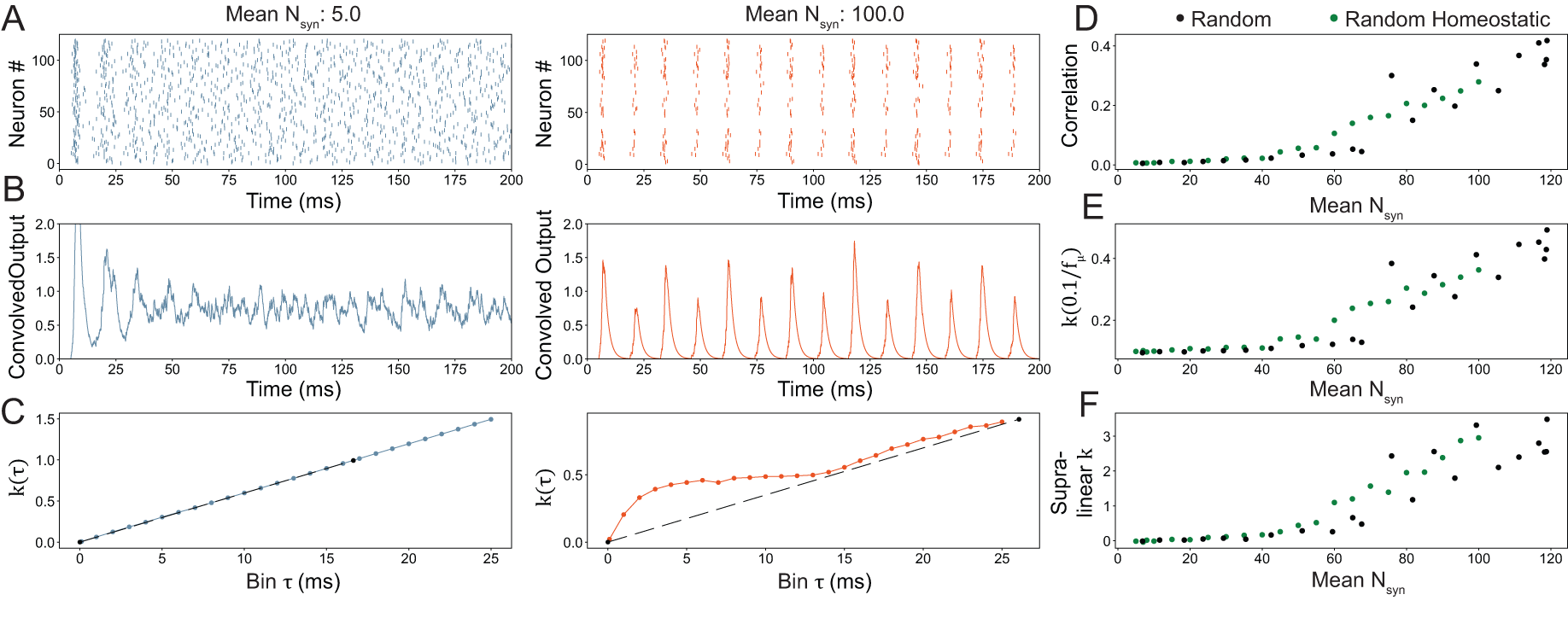

Supplement: Figure 1-4 — Keeping the number of synaptic inputs homogeneous does not change measured synchrony. In these simulations, each postynaptic neuron randomly chooses Nsyn presynaptic neurons to receive input from. Therefore, each neuron receives the same number of synapses. A,B&C show the same information as in main Figure 1, but for the simulations with equal synaptic inputs. D,E&F show the synchrony measures. In black are the same data as shown in main Figure 1. In green are the simulations with equal synapse numbers. Download Figure 1-4, TIF file. [file eneuro-12-ENEURO.0097-25.2025-s005.tif]

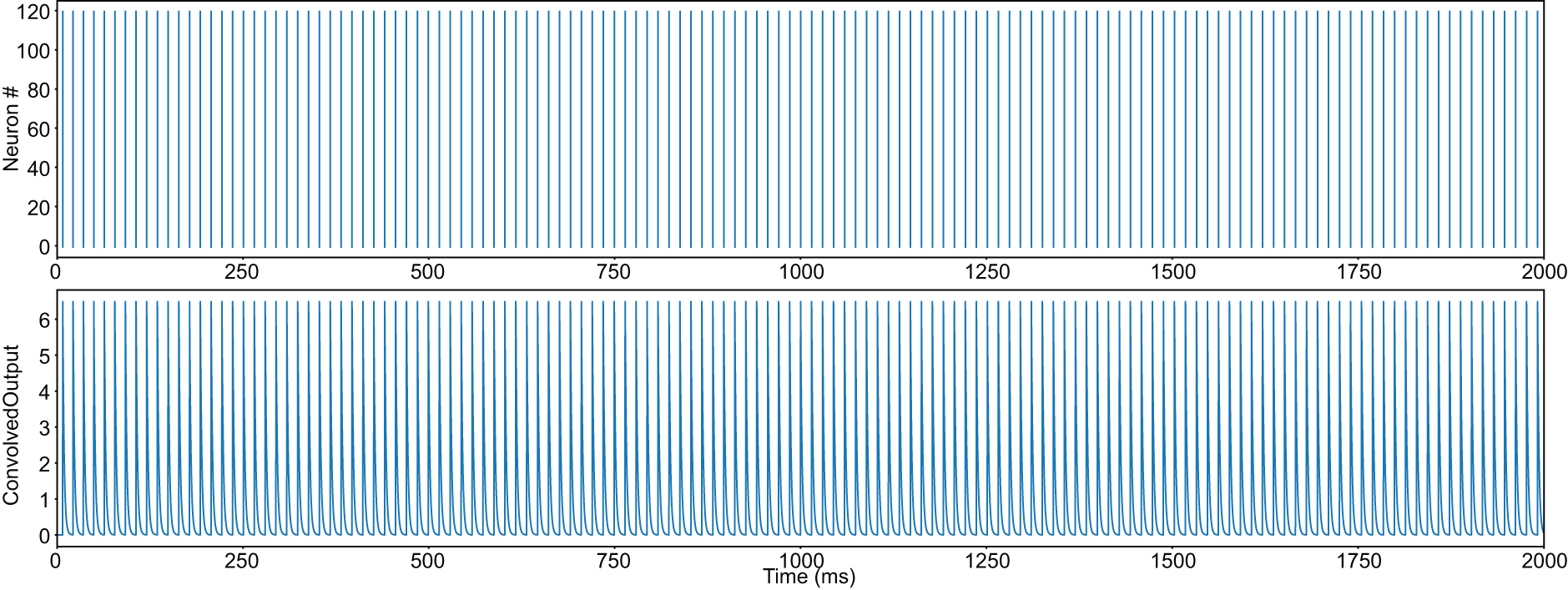

Supplement: Figure 1-5 — Identical neurons with identical number of incoming synapses are exhibit perfectly synchronous activity. Each neuron receives input from 8 randomly chosen other neurons in the ring network. Each neuron receives a constant somatic current injection of 300 pA. Under these conditions all neurons fire at exactly the same time, there their correlation coefficient is 1, their k(0.1fμ) is also 1 and their supralinear k is 8.47. Download Figure 1-5, TIF file. [file eneuro-12-ENEURO.0097-25.2025-s006.tif]

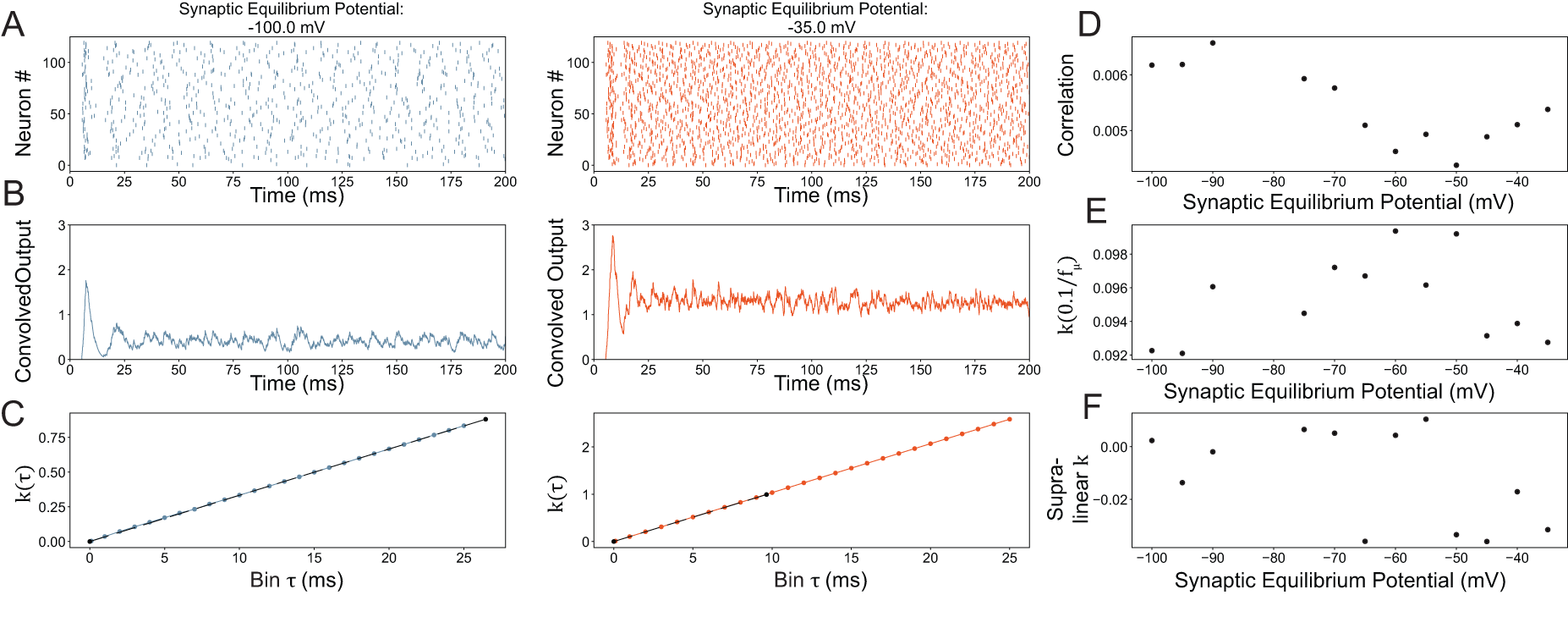

Supplement: Figure 1-6 — Varying the reversal potential of the inhibitory synapses in the ring model does not affect synchrony measures. For reference, the equilibrium potential in the baseline model is -70 mV. Download Figure 1-6, TIF file. [file eneuro-12-ENEURO.0097-25.2025-s007.tif]

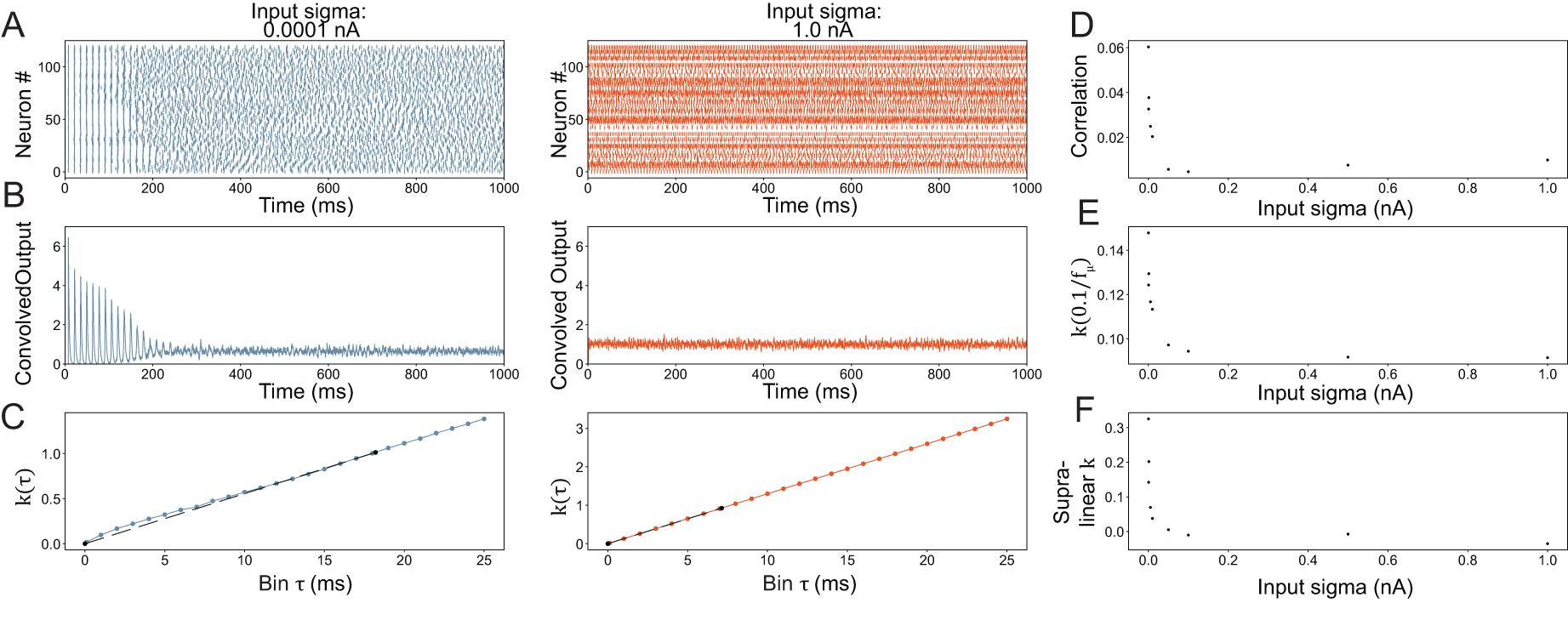

Supplement: Figure 1-7 — A network with low variance input current is initially synchronous but desynchronizes over time. For reference, the sigma of the input current in the baseline model is 0.3 nA. Download Figure 1-7, TIF file. [file eneuro-12-ENEURO.0097-25.2025-s008.tif]

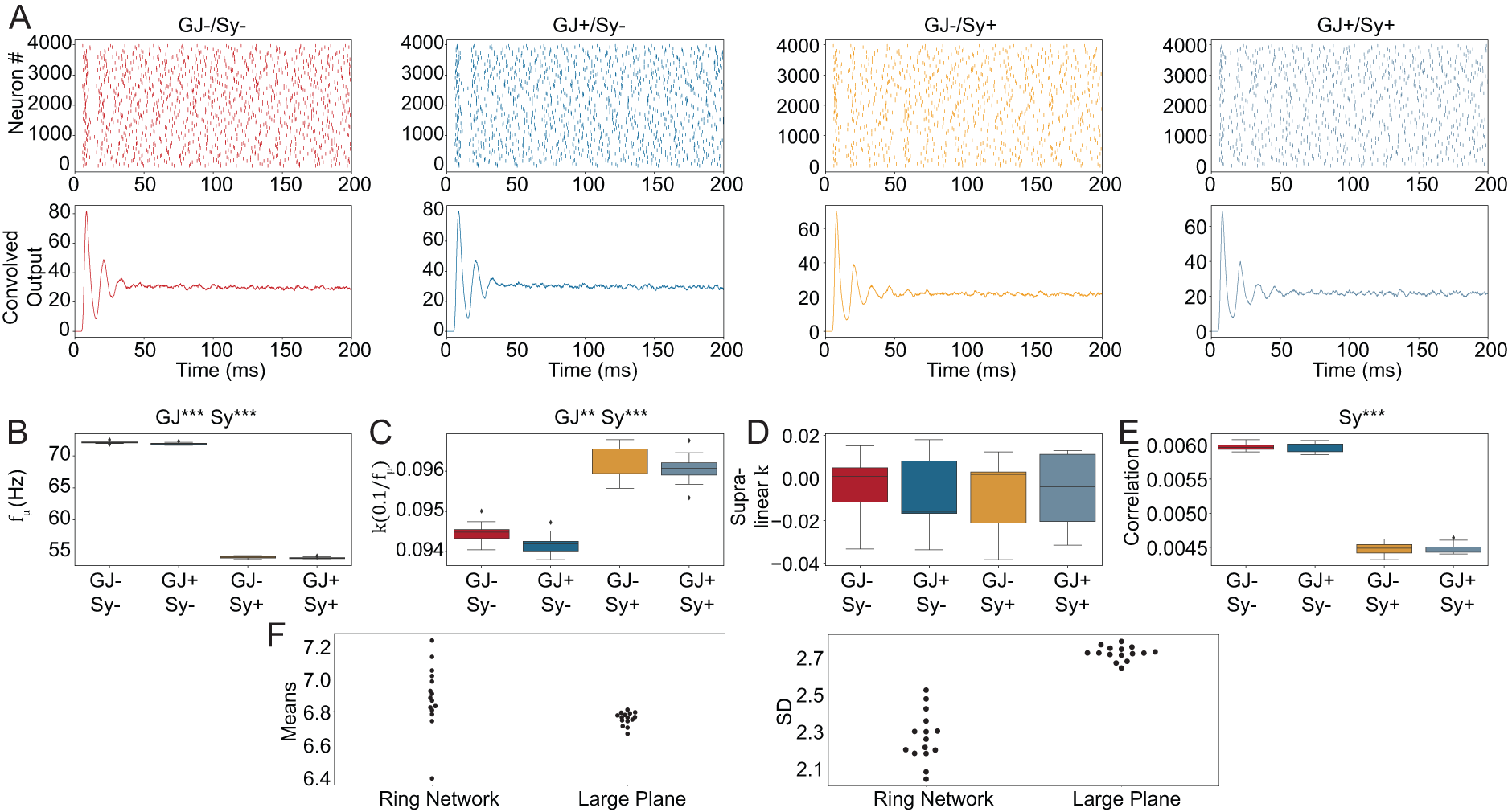

Supplement: Figure 2-1 — Large PV + IN plane network. 4000 neurons were randomly distributed on a rectangular plane. A The spike raster plots show only every 40th neuron. Raster plot and convolved output show that the state is asynchronous. B Average frequency of all neurons in the network. Two-way ANOVA: Interaction: F = 1.893, p = 0.1743, Main effects: GJ, F = 14.0517, p < 0.001; Sy, F = 164105.4068, p < 0.001. C-D The synchrony measures. Two-way ANOVA for k(0.1fμ): Interaction: F = 0.8884, p = 0.35, Main effects: GJ, F = 8.0496, p < 0.01; Sy, F = 540.5753, p < 0.001. Two-way ANOVA for Supralinear had no significant effects. Two-way ANOVA for Correlation: Interaction: F = 0.191, p = 0.6638, Main effects: GJ, F = 0.2828, p = 0.597; Sy, F = 6841.9982, p < 0.001. GJ, gap junction; Sy, synapse. F shows the Mean number of synapses and SD for each sample of the ring network from main Figure 2 and the large plane network of the present figure. The average mean number of synapses is was 6.89 in the ring network and 6.76 in the large plane network. The average SD in the ring network was 2.28 and in the large plane network it was 2.73. Download Figure 2-1, TIF file. [file eneuro-12-ENEURO.0097-25.2025-s009.tif]

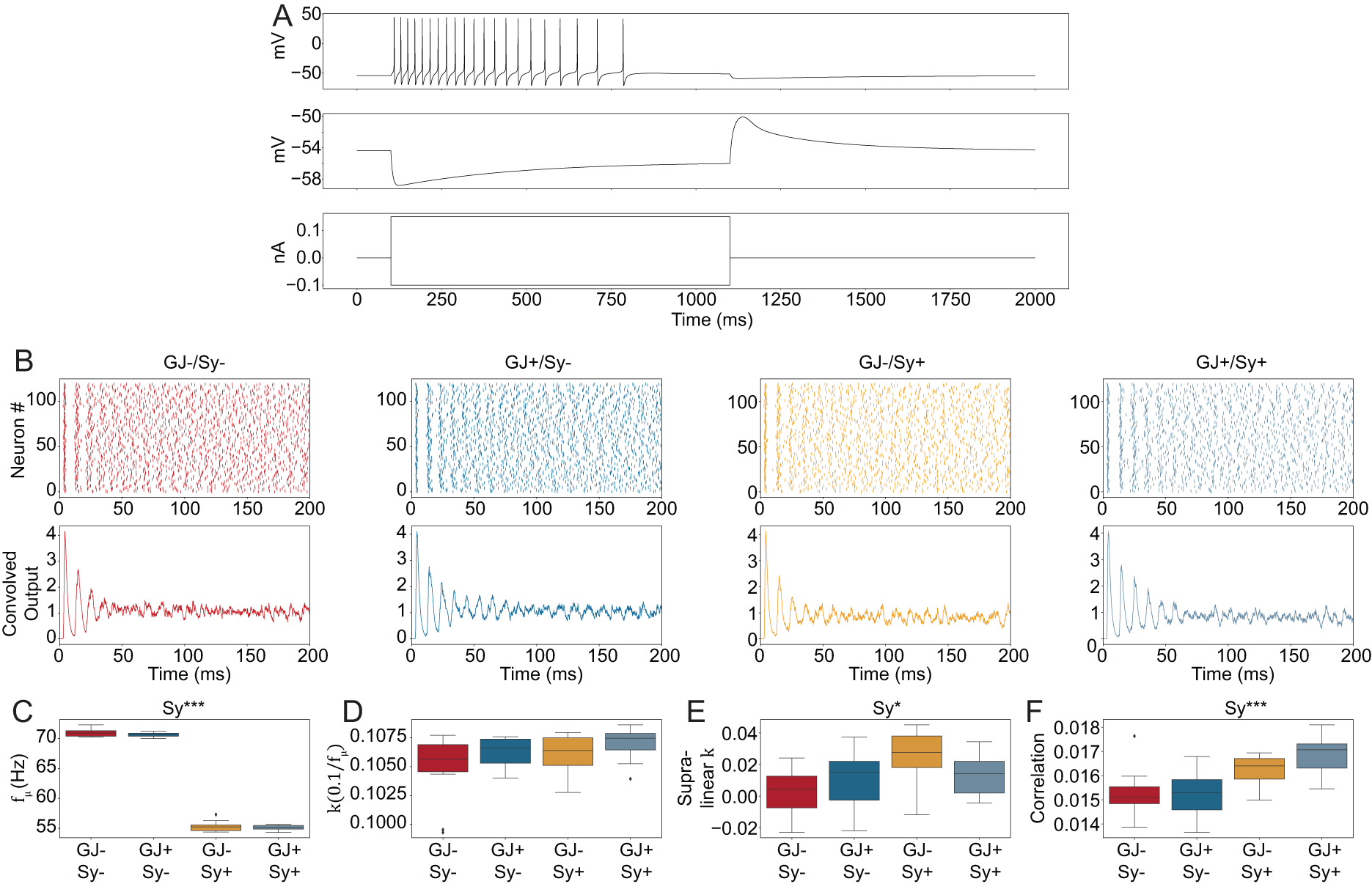

Supplement: Figure 2-2 — Ring network simulations with PV+ INs containing Ih current. A Voltage response of the modified PV+ IN with Ih added. Top: voltage response to depolarizing current step. Middle: response to hyperpolarizing current step with the slow depolarization characteristic for Ih. Bottom: The current injections that induce the voltage responses above. B Spike raster plot and convolved output show an asynchronous state. C Average frequency of all neurons in the network. Twoway ANOVA: Interaction: F = 0.0926, p = 0.7626, Main effects: GJ, F = 1.8648, p = 0.1805; Sy, F = 5884.6182, p < 0.001. D-F The synchrony measures. Two-way ANOVA for k(0.1fμ) had no significant results. Two-way ANOVA for Supralinear k: Interaction: F = 4.0058, p = 0.0529, Main effects: GJ, F = 0.1206, p = 0.7304; Sy, F = 6.8143, p < 0.05. Two-way ANOVA for Correlation: Interaction: F = 1.2169, p = 0.2773, Main effects: GJ, F = 1.0703, p = 0.3078; Sy, F = 21.5984, p < 0.001. Download Figure 2-2, TIF file. [file eneuro-12-ENEURO.0097-25.2025-s010.tif]

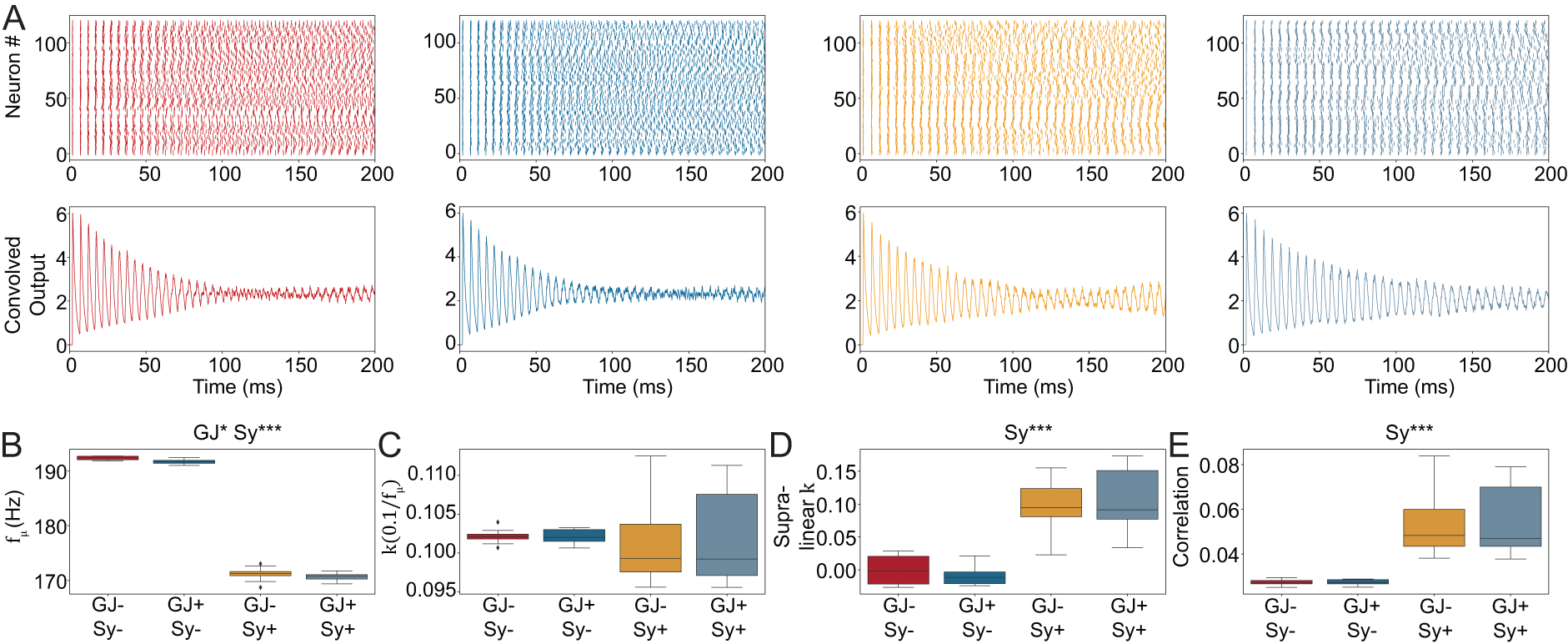

Supplement: Figure 2-3 — Ring network simulation with strong input current resulting in high average frequency. A Spike raster plot and convolved output show that some synchronous oscillations are larger when chemical synapses are added. However, this synchronous state is different from the one in Figure 1, where cells become silent during a cycle and furthermore does not reach the magnitude. B Average frequency of all neurons in the network. Two-way ANOVA: Interaction: F = 0.1127, p = 0.7391, Main effects: GJ, F = 5.3695, p < 0.05; Sy, F = 7474.6760, p < 0.001. C-E The synchrony measures. Two-way ANOVA for k(0.1fμ) had no significant results. Two-way ANOVA for Supralinear k: Interaction: F = 0.9549, p = 0.3350, Main effects: GJ, F = 0.0117, p = 0.9145; Sy, F = 97.124, p < 0.001. Two-way ANOVA for Correlation: Interaction: F = 0.0059, p = 0.9393, Main effects: GJ, F = 0.0137, p = 0.9076; Sy, F = 60.3379, p < 0.001. Download Figure 2-3, TIF file. [file eneuro-12-ENEURO.0097-25.2025-s011.tif]

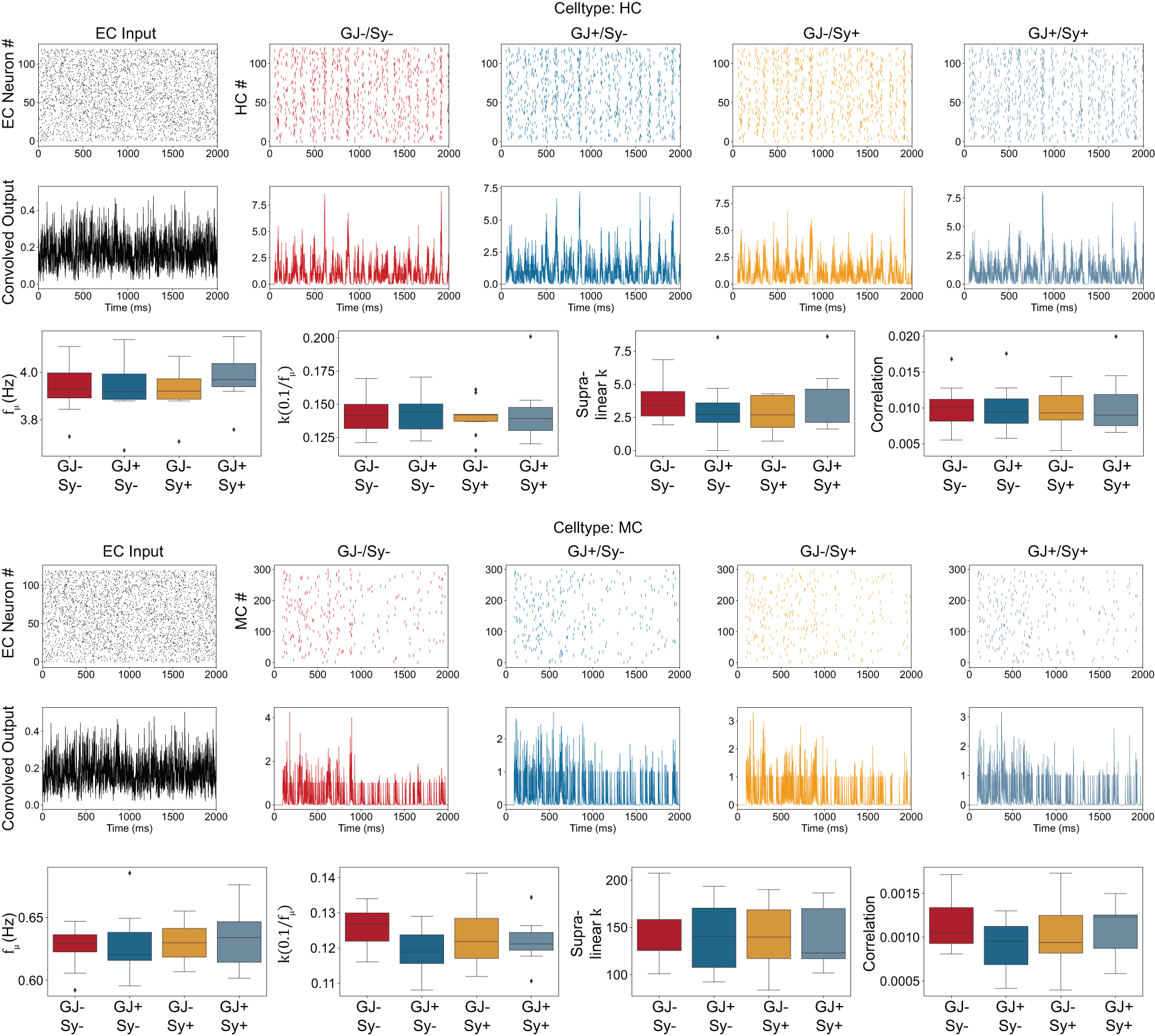

Supplement: Figure 4-1 — Results for the HC (top) and MC (bottom), which were simulated for Figure 4 but not shown. Two-way ANOVA was done for all of the box plots but showed no significant result for any of them. Download Figure 4-1, TIF file. [file eneuro-12-ENEURO.0097-25.2025-s012.tif]

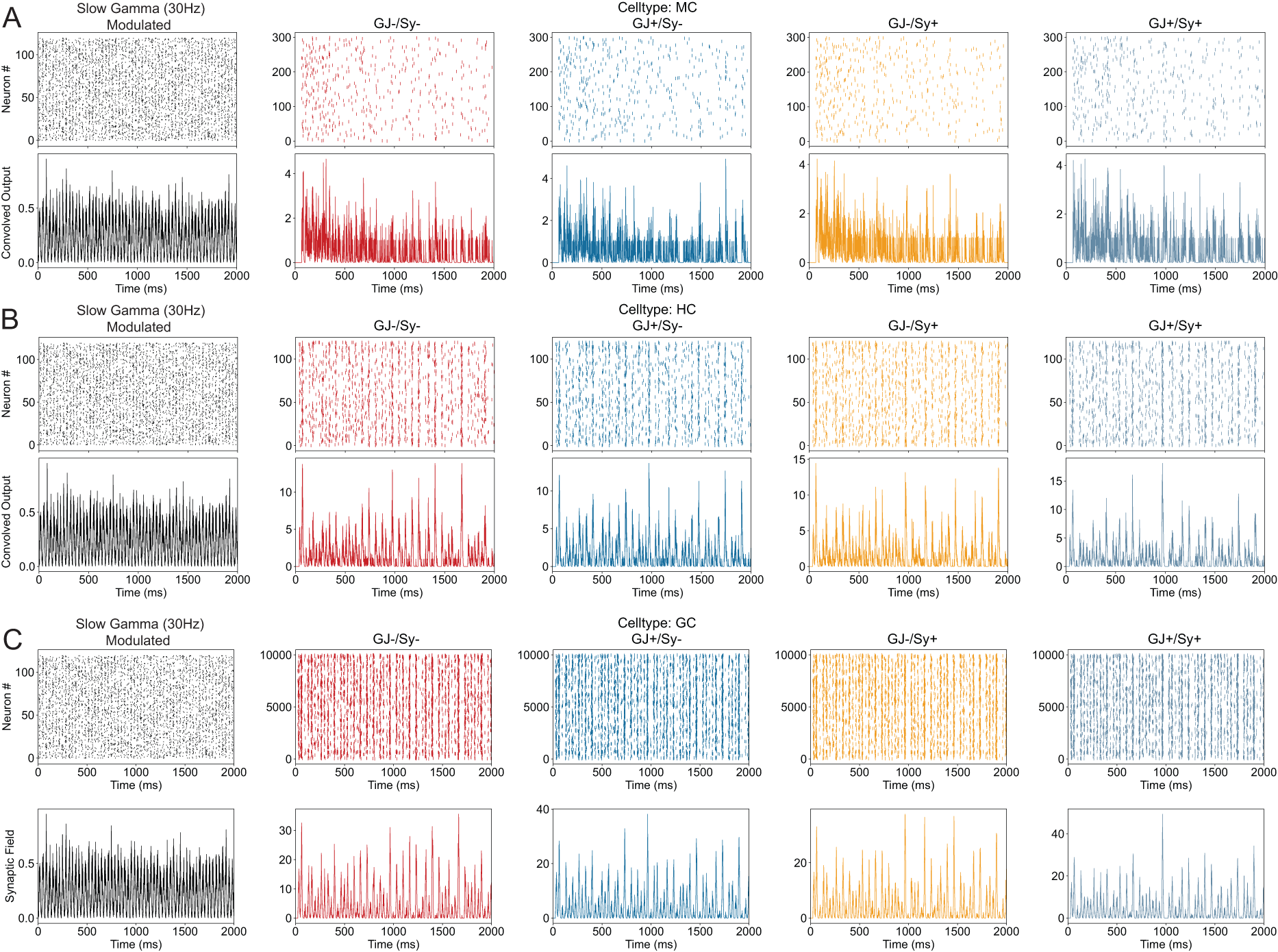

Supplement: Figure 5-1 — Cell types that were simulated in the slow gamma condition in Figure 5 but not shown. Boxplots were omitted because none of them was significant. Download Figure 5-1, TIF file. [file eneuro-12-ENEURO.0097-25.2025-s013.tif]

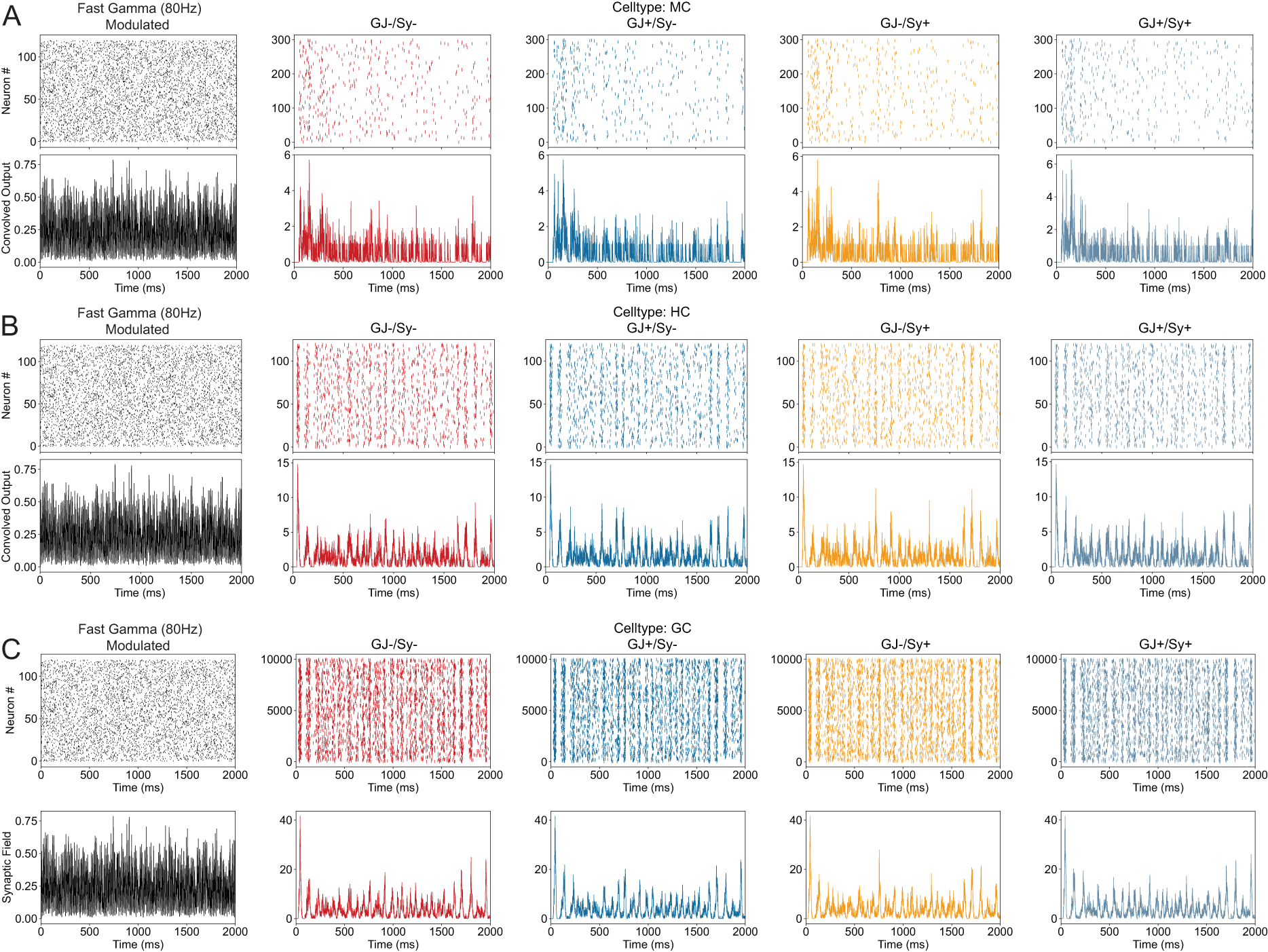

Supplement: Figure 5-2 — Cell types that were simulated in the fast gamma condition in Figure 5 but not shown. Boxplots were omitted because none of them was significant with the sole exception of a significant increase of average HC frequency in the fast gamma condition when synaptic connectivity is added in the PV+ ring network: F = 5.322589, p < 0.05. Download Figure 5-2, TIF file. [file eneuro-12-ENEURO.0097-25.2025-s014.tif]
