# Supplementary material for: Recurrent Interneuron Connectivity Does Not Support Synchrony in a Biophysical Dentate Gyrus Model
Source: eNeuro. 2025 Apr 18;12(4):ENEURO.0097-25.2025. doi: 10.1523/ENEURO.0097-25.2025 (PMC12017885; doi:10.1523/ENEURO.0097-25.2025)
Supplement: Table 4-1 — Parameters that are equal in all four neuron types. ccanl, intracellular calcium accumulation with exponential decay to baseline. Download Table 4-1, DOCX file. [file eneuro-12-ENEURO.0097-25.2025-s015.docx]

Supp. Table. 4 - 1: Parameters that are equal in all four neuron types. ccanl, intracellular calcium accumulation with exponential decay to baseline.

| Location | Mechanism | Parameter | Value |
| --- | --- | --- | --- |
| All | ccanl | catau | 10.0 |
| All | ccanl | caiinf | 0.000005 |
| All | Membrane | ekf | -90.0 |
| All | Membrane | ek | -90.0 |
| All | Membrane | elca | 130.0 |
| All | Membrane | esk | -90 |
