# Supplementary material for: Recurrent Interneuron Connectivity Does Not Support Synchrony in a Biophysical Dentate Gyrus Model
Source: eNeuro. 2025 Apr 18;12(4):ENEURO.0097-25.2025. doi: 10.1523/ENEURO.0097-25.2025 (PMC12017885; doi:10.1523/ENEURO.0097-25.2025)
Supplement: Table 4-2 — The intrinsic parameters of the granule cell model. borgka, Borg-Grahamgeneric A-type potassium channel; cagk, a voltage dependent calcium activated potassium channel; gskch, a nonvoltage-dependent calcium-activated potassium channel; ichan2, a mechanism combining Hodgkin-Huxley style sodium and potassium conductances; lca, an L-type calcium channel;ore, An N-type calcium channel (nca) was used in all cell types except for HIPP cells. T-Type calcium channels (cat) were used only in granule cells. Persistently modified h-channels (hyperde3) were used in HIPP and Mossy cells. Download Table 4-2, DOCX file. [file eneuro-12-ENEURO.0097-25.2025-s016.docx]

Supp. Table. 4 - 2: The intrinsic parameters of the granule cell model. borgka, Borg-Graham generic A-type potassium channel; cagk, a voltage dependent calcium activated potassium channel; gskch, a non-voltage-dependent calcium-activated potassium channel; ichan2, a mechanism combin- ing Hodgkin-Huxley style sodium and potassium conductances; lca, an L-type calcium channel;ore, An N-type calcium channel (nca) was used in all cell types except for HIPP cells. T-Type calcium channels (cat) were used only in granule cells. Persistently modified h-channels (hyperde3) were used in HIPP and Mossy cells.

| Location | Mechanism | Parameter | Value |
| --- | --- | --- | --- |
| All | ichan2 | el | -70.0 |
| All | Membrane | Ra | 210.0 |
| All | Membrane | enat | 45.0 |
| All | Membrane | eks | -90.0 |
| All | Membrane | etca | 130.0 |
| Soma | Membrane | cm | 1.0 |
| Soma | ichan2 | gnatbar | 0.12 |
| Soma | ichan2 | gkfbar | 0.016 |
| Soma | ichan2 | gksbar | 0.006 |
| Soma | ichan2 | gl | 0.00004 |
| Soma | borgka | gkabar | 0.012 |
| Soma | nca | gncabar | 0.002 |
| Soma | lca | glcabar | 0.005 |
| Soma | cat | gcatbar | 0.000037 |
| Soma | gskch | gskbar | 0.001 |
| Soma | cagk | gkbar | 0.0006 |
| GCLD | Membrane | cm | 1.0 |
| GCLD | ichan2 | gnatbar | 0.018 |
| GCLD | ichan2 | gkfbar | 0.004 |
| GCLD | ichan2 | gksbar | 0.006 |
| GCLD | ichan2 | gl | 0.00004 |
| GCLD | nca | gncabar | 0.003 |
| GCLD | lca | glcabar | 0.0075 |
| GCLD | cat | gcatbar | 0.000075 |
| GCLD | gskch | gskbar | 0.0004 |
| GCLD | cagk | gkbar | 0.0006 |
| PROXD | Membrane | cm | 1.6 |
| PROXD | ichan2 | gnatbar | 0.013 |
| PROXD | ichan2 | gkfbar | 0.004 |
| PROXD | ichan2 | gksbar | 0.006 |
| PROXD | ichan2 | gl | 0.000063 |
| PROXD | nca | gncabar | 0.001 |
| PROXD | lca | glcabar | 0.0075 |
| PROXD | cat | gcatbar | 0.00025 |
| PROXD | gskch | gskbar | 0.0002 |
| PROXD | cagk | gkbar | 0.001 |
| MIDD | Membrane | cm | 1.6 |
| MIDD | ichan2 | gnatbar | 0.008 |
| MIDD | ichan2 | gkfbar | 0.001 |
| MIDD | ichan2 | gksbar | 0.006 |
| MIDD | ichan2 | gl | 0.000063 |
| MIDD | nca | gncabar | 0.001 |
| MIDD | lca | glcabar | 0.0005 |
| MIDD | cat | gcatbar | 0.0005 |
| MIDD | gskch | gskbar | 0.0 |
| MIDD | cagk | gkbar | 0.0024 |
| DD | Membrane | cm | 1.6 |
| DD | ichan2 | gnatbar | 0.0 |
| DD | ichan2 | gkfbar | 0.001 |
| DD | ichan2 | gksbar | 0.008 |
| DD | ichan2 | gl | 0.000063 |
| DD | nca | gncabar | 0.001 |
| DD | lca | glcabar | 0.0 |
| DD | cat | gcatbar | 0.001 |
| DD | gskch | gskbar | 0.0 |
| DD | cagk | gkbar | 0.0024 |
