# Supplementary material for: Recurrent Interneuron Connectivity Does Not Support Synchrony in a Biophysical Dentate Gyrus Model
Source: eNeuro. 2025 Apr 18;12(4):ENEURO.0097-25.2025. doi: 10.1523/ENEURO.0097-25.2025 (PMC12017885; doi:10.1523/ENEURO.0097-25.2025)
Supplement: Table 4-5 — The intrinsic parameters of the HIPP cell model. Download Table 4-5, DOCX file. [file eneuro-12-ENEURO.0097-25.2025-s019.docx]

Supp. Table. 4 - 5: The intrinsic parameters of the HIPP cell model.

| Location | Mechanism | Parameter | Value |
| --- | --- | --- | --- |
| All | ichan2 | el | -70.45 |
| All | borgka | gkabar | 0.0008 |
| All | lca | glcabar | 0.0015 |
| All | gskch | gskbar | 0.003 |
| All | cagk | gkbar | 0.003 |
| All | hyperde3 | ghyfbar | 0.000015 |
| All | hyperde3 | ghysbar | 0.000015 |
| All | Membrane | Ra | 100.0 |
| All | Membrane | enat | 55.0 |
| All | Membrane | ehyf | -40 |
| All | Membrane | ehys | -40 |
| Soma | ichan2 | gnatbar | 0.2 |
| Soma | ichan2 | gkfbar | 0.006 |
| Soma | ichan2 | gl | 0.000036 |
| Soma | Membrane | cm | 1.1 |
| PROXD | ichan2 | gnatbar | 0.2 |
| PROXD | ichan2 | gkfbar | 0.006 |
| PROXD | ichan2 | gl | 0.000036 |
| PROXD | Membrane | cm | 1.1 |
| MIDD | ichan2 | gnatbar | 0.0 |
| MIDD | ichan2 | gkfbar | 0.0 |
| MIDD | ichan2 | gl | 0.000036 |
| MIDD | Membrane | cm | 1.1 |
| DD | ichan2 | gnatbar | 0.0 |
| DD | ichan2 | gkfbar | 0.0 |
| DD | ichan2 | gl | 0.000036 |
| DD | Membrane | cm | 1.1 |
