# Supplementary material for: Recurrent Interneuron Connectivity Does Not Support Synchrony in a Biophysical Dentate Gyrus Model
Source: eNeuro. 2025 Apr 18;12(4):ENEURO.0097-25.2025. doi: 10.1523/ENEURO.0097-25.2025 (PMC12017885; doi:10.1523/ENEURO.0097-25.2025)
Supplement: Table 4-6 — The parameters of the synaptic connections. Columns: Pre, the presynaptic population; Post, the postsynaptic populations; N Target, number of nearby neurons that a neuron can connect to; Dendrite, dendrite of the postsynaptic neuron that is targeted by the synapse; Divergence, number of postsynaptic neurons that each presynaptic neuronc chooses randomly from the population defined by N Target; τ1, Decay time constant; τfacil, facilitation time constant of the Tsodyks-Markam dynamics. Download Table 4-6, DOCX file. [file eneuro-12-ENEURO.0097-25.2025-s020.docx]

Supp. Table. 4 - 6: The parameters of the synaptic connections. Columns: Pre, the presynaptic population; Post, the postsynaptic populations; N Target, number of nearby neurons that a neuron can connect to; Dendrite, dendrite of the postsynaptic neuron that is targeted by the synapse; Divergence, number of postsynaptic neurons that each presynaptic neuronc chooses randomly from the population defined by N Target; *τ*_1_, Decay time constant; *τ_facil_*, facilitation time constant of the Tsodyks-Markam dynamics.

| Pre | Post | N Target | Dendrite | Divergence | *τ*_1_ | *τfacil* | U | *τrec* | e | Threshold | delay | weight |
| --- | --- | --- | --- | --- | --- | --- | --- | --- | --- | --- | --- | --- |
| PP | GC | - | MIDD | 1 | 1 | 1 | 1 | 1 | 1 | 1 | 1 | 1 |
| PP | BC | - | DDEND | 1 | 1 | 1 | 1 | 1 | 1 | 1 | 1 | 1 |
| GC | MC | 60 | PROXD | 1 | 7.6 | 500 | 0.1 | 0 | 0 | 10 | 1.5 | 2e-2 |
| GC | BC | 40 | PROXD | 1 | 8.7 | 500 | 0.1 | 0 | 0 | 10 | 0.8 | 2.5e-2 |
| GC | HC | 120 | PROXD | 1 | 8.7 | 500 | 0.1 | 0 | 0 | 10 | 1.5 | 2.5e-2 |
| MC | MC | 120 | PROXD | 3 | 2.2 | 0 | 1 | 0 | 0 | 10 | 2 | 5e-4 |
| MC | BC | 60 | PROXD | 1 | 2 | 0 | 1 | 0 | 0 | 10 | 3 | 3e-4 |
| MC | HC | 100 | MIDD | 2 | 6.2 | 0 | 1 | 0 | 0 | 10 | 3 | 2e-4 |
| BC | GC | 2800 | SOMA | 400 | 20 | 0 | 1 | 0 | -70 | 10 | 0.85 | 1.2e-3 |
| BC | MC | 140 | PROXD | 3 | 3.3 | 0 | 1 | 0 | -70 | 10 | 1.5 | 1.5e-3 |
| BC | BC | - | PROXD | - | 1.8 | 0 | 1 | 0 | -10 | 10 | 0.8 | 7.6e-3 |
| HC | GC | 10000 | DD | 640 | 20 | 0 | 1 | 0 | -70 | 10 | 3.8 | 6e-3 |
| HC | MC | 300 | MIDD | 4 | 6 | 0 | 1 | 0 | -70 | 10 | 1 | 1.5e-3 |
| HC | BC | 120 | DDEND | 4 | 5.8 | 0 | 1 | 0 | -70 | 10 | 1.6 | 5e-4 |
